# Intrapulmonary Autoantibodies to HSP72 Are Associated with Improved Outcomes in IPF

**DOI:** 10.1155/2019/1845128

**Published:** 2019-04-11

**Authors:** Ross Mills, Abhinav Mathur, Lisa M. Nicol, Jeremy J. Walker, Alexander A. Przybylski, Alison C. Mackinnon, Sarah E. M. Howie, William A. H. Wallace, Ian Dransfield, Nik Hirani

**Affiliations:** Centre for Inflammation Research at the QMRI, University of Edinburgh, Edinburgh, UK

## Abstract

**Rationale:**

Idiopathic pulmonary fibrosis (IPF) is a progressive fibrotic interstitial lung disease, with high mortality. Currently, the aetiology and the pathology of IPF are poorly understood, with both innate and adaptive responses previously being implicated in the disease pathogenesis. Heat shock proteins (Hsp) and antibodies to Hsp in patients with IPF have been suggested as therapeutic targets and prognostic biomarkers, respectively. We aimed to study the relationship between the expression of Hsp72 and anti-Hsp72 antibodies in the BAL fluid and serum Aw disease progression in patients with IPF.

**Methods:**

A novel indirect ELISA to measure anti-Hsp72 IgG was developed and together with commercially available ELISAs used to detect Hsp72 IgG, Hsp72 IgGAM, and Hsp72 antigen, in the serum and BALf of a cohort of IPF (*n* = 107) and other interstitial lung disease (ILD) patients (*n* = 66). Immunohistochemistry was used to detect Hsp72 in lung tissue. The cytokine expression from monocyte-derived macrophages was measured by ELISA.

**Results:**

Anti-Hsp72 IgG was detectable in the serum and BALf of IPF (*n* = 107) and other ILDs (*n* = 66). Total immunoglobulin concentrations in the BALf showed an excessive adaptive response in IPF compared to other ILDs and healthy controls (*p* = 0.026). Immunohistochemistry detection of C4d and Hsp72 showed that these antibodies may be targeting high expressing Hsp72 type II alveolar epithelial cells. However, detection of anti-Hsp72 antibodies in the BALf revealed that increasing concentrations were associated with improved patient survival (adjusted HR 0.62, 95% CI 0.45-0.85; *p* = 0.003). *In vitro* experiments demonstrate that anti-Hsp72 complexes stimulate macrophages to secrete CXCL8 and CCL18.

**Conclusion:**

Our results indicate that intrapulmonary anti-Hsp72 antibodies are associated with improved outcomes in IPF. These may represent natural autoantibodies, and anti-Hsp72 IgM and IgA may provide a beneficial role in disease pathogenesis, though the mechanism of action for this has yet to be determined.

## 1. Introduction

Idiopathic pulmonary fibrosis (IPF) is the most common interstitial lung disease (ILD) with an estimated prevalence of 10-60 cases per 100,000 people worldwide [1]. IPF is characterised by irreversible and progressive fibrosis, with a specific histological feature, namely, usual interstitial pneumonia (UIP). Compared to other ILDs, IPF has the poorest prognosis but a highly variable clinical course [2–4]. A multidisciplinary approach to diagnosis is required, and a confident diagnosis may require patients to undergo lung biopsy. Currently, the mechanisms underpinning disease progression are poorly understood, but it is thought that an unidentified inflammatory incident may lead to a continuous and disruptive immune response with excessive alveolar epithelial cell (AEC) death and aberrant reepithelisation with type II AECs [5].

A role for IgG in IPF pathology was originally postulated when detection of complement activation through the classical pathway was seen in the pleural space [6] and immune complexes were shown in the alveolar fluid of IPF patients [7]. Both humoral and cell-mediated immunity have been implicated in the pathogenesis of IPF. Heightened circulating concentrations of CXCL13 [8] and lung parenchymal proliferative B cell aggregates with dendritic cells forming germinal centers [9, 10] indicate the presence of an active humoral response. This has led to the hypothesis that B cells contribute to the pathogenesis UIP and is the rationale for B cell-targeted therapies in IPF [11]. It has been further hypothesised that this humoral response may be autoimmune in nature. Previous studies have demonstrated that T cells isolated from IPF patients can proliferate in response to lung-derived autoantigens indicating a breakdown in self-tolerance [12–15]. Further to this, it was observed that CD4^+^ T cells only proliferated in response to protein samples prepared from IPF lung tissue and not healthy tissue [15]. Circulating autoreactive IgG, against a 70-90 kDa alveolar epithelial cell (AEC) antigen, was first detected in IPF patients by Wallace et al. [16, 17]. More recently, circulating antibodies to Hsp72 were shown to be associated with poorer outcomes in IPF [14] but have not yet been validated in independent cohorts. A number of other autoreactive IgG antibodies have been described that similarly target AEC antigens including annexin-1 [18] and cytokeratins-8 and -19 [19, 20], periplakin [21], and vimentin [22] BPIFB1 [23]. Certain autoantibodies detected in IPF have also been described as having functional effects, with anti-Hsp72 IgG from IPF patients inducing the secretion of CXCL8 and expression of CD69 in monocytes [14]. The transfer of autoantibodies to BPIFB1 into *Rag^−/−^* mice did not result in ILD. However, the transfer of BPIFB1-specific CD4^+^ cells led to spontaneous ILD induction which was not observed in mice with intact immune systems [23]. This suggests that a break in T cell tolerance could lead to the development of ILD. However, others have suggested that autoantibodies in ILD may be epiphenomenal [24].

The presence of IgG autoantibodies is generally considered to represent a breakdown of self-tolerance and is associated with pathological consequences. Alternatively, these antibodies may instead represent IgG “natural” autoantibodies that have been described in healthy individuals [25] and may have a role in maintaining homeostasis. Natural autoantibodies are recognised as being polyreactive with low affinities [26] and recognise highly conserved molecules [27]. In COPD, natural autoantibodies have been seen to target similar antigens as those previously detected in IPF, including cytokeratin-18, as well as disease-specific autoantibodies such as against collagen 5 [28]. Therefore, there is some evidence for the presence of natural autoantibodies in ILD. The compartment, circulating versus tissue, in which these postulated natural IgGs are expressed, may provide insights into their role. In addition, the presence of autoantibodies in the serum of IPF patients may provide a novel means of prognosticating IPF patients. Detection of anti-Hsp72 [14] and anti-periplakin IgG [21] have been (independently) linked to poorer outcomes in IPF; however, anti-Hsp72 IgG has yet to be validated and anti-periplakin IgG was undetectable in a different cohort of IPF patients [29]. We hypothesise that elevated anti-Hsp72 IgG will associate with IPF disease progression and will induce immune cells to promote a fibrotic response.

## 2. Methods

### 2.1. Patient Demographic and Ethical Consent

Ethical permission was granted from the NHS Lothian Research ethics board (LREC 07/S1102/20 06/S0703/53). Participants were incident cases attending the Edinburgh Lung Fibrosis Clinic. Serum samples were obtained at first presentation to the clinic, and BAL was obtained within the first 12 months of first presentation, patient demographics used in this study are in Table 1. All diagnoses of ILD were made by multidisciplinary consensus according to ATS/ERS consensus criteria [30]. “Other ILD” comprises a group of non-IPF ILD patients with the disease breakdown of 13 patients with hypersensitivity pneumonitis (HP), 11 patients with idiopathic nonspecific interstitial pneumonia (NSIP), and 42 patients with “unclassifiable” disease. “Unclassifiable” ILD refers to disease that (i) was idiopathic, (ii) did not fulfil CT criteria for definite IPF or possible IPF and have a radiological pattern that is compatible but not diagnostic of at least one of the recognised forms of interstitial pneumonia, and (iii) is not biopsied. These unclassifiable entities are further divided into those with and without fibrosis based on CT imaging [4]. Serum and bronchoalveolar lavage (BAL) samples were taken with permission from 157 ILD patients; IPF disease progression was defined as a decrease of ≥10% in lung function as measured by vital capacity (VC) or pulmonary-related death in the twelve months postsampling [2, 3]. “Control” bronchoalveolar lavage and serum were obtained from subjects that required bronchoscopy for isolated haemoptysis but had no evidence of lung disease based on CT imaging and are referred to as “healthy controls.”

### 2.2. Immunoassays

Commercial anti-Hsp72 IgG assays were not available at the time of study; thus, anti-Hsp72 IgG was measured using an in-house optimised indirect ELISA, using the ILD patient serum as a source for the anti-Hsp72 IgG standard. The serum from 5 patients was pooled to generate the anti-Hsp72 IgG standard and was determined using the method described below and identified by the increased 450 nm OD readings measured. To briefly describe, 100 ng/ml recombinant (r)Hsp72 (SignalChem, H34-54H) was incubated overnight at 4°C in an ELISA plate. Between each of the following steps, 3 washes were made using PBS with 0.1%Tween. ELISA plates were blocked using RD (R&D DY995, 10x reagent diluent diluted 1 : 10 in PBS). Patient samples were optimised for the assay; serum samples were diluted 1 : 1000 and BALf samples 1 : 100 in RD and incubated for one hour (samples outside the standards were reanalysed with more appropriate dilutions as required). Detection of anti-Hsp72 IgG was performed using a biotin-conjugated anti-human IgG (Vector Laboratories, BA-3000), incubated for 2 hours. HRP-conjugated streptavidin (R&D Systems, sourced from other commercial ELISA kits) was incubated at the appropriate concentrations, and TMB-HRP reactions were measured using a BioTek Synergy H1 Hybrid Plate Reader.

ELISAs for CXCL8 (DY208), Hsp72 (DY1663), and CCL18 (DY394) were sourced from R&D (Abingdon, UK). ELISA for total IgG (88-50550), total IgA (88-50600), and total IgM (88-50620) was sourced from Affymetrix eBioscience (High Wycombe, UK). ELISAs for anti-Hsp72 IgGAM (ADI-EKS-750) were sourced from Enzo Life Sciences (Exeter, UK). These ELISAs were used following the manufacturers' protocol.

### 2.3. Protein G Isolation of IPF Patient IgG

BALf IgG from 6 IPF patients was purified using Protein G (Thermo Fisher Scientific, #20398). For each patient, 4 ml of BALf was diluted 1 : 1 with Protein G binding buffer (Thermo Fisher Scientific, #21011) and allowed to gravity filter through Protein G agarose. Purified IgG was eluted using 2 ml elution buffer (Thermo Fisher Scientific, #21004), and the pH was stabilised with 100 *μ*l Tris (pH 8).

### 2.4. MDM Cell Culture

PBMCs were isolated from whole blood using a standard Ficoll-Paque isolation method. Whole blood was diluted 1 : 1 with sterile PBS. Diluted whole blood was then layered over Ficoll at a 3 : 1 ratio of volume (3 ml of blood per 1 ml of Ficoll). Samples were then centrifuged at 1500g for 20 minutes without braking. After centrifugation, cells were collected, washed in PBS, and counted prior to monocyte isolation.

CD14^+^/CD16^−^, CD14^−^/CD16^+^, and CD14^+^/CD16^+^ monocytes were negatively selected from the PBMCs using a Pan-monocyte isolation kit (Miltenyi Biotec, 130-096-537) following the manufacturer's protocol. Isolated monocytes were differentiated into MDMs by culturing them for 4 days in a 96-well culture plate in IMDM supplemented with 10% FBS, 1% L-glutamine, 100 *μ*g/ml penicillin, 100 IU/ml streptomycin, 2% AB serum, and 5 ng/ml M-CSF. Following 4 days of culture, MDMs were treated with the following conditions for 24 hours: untreated, 100 ng/ml Hsp72 (Sino Biological, 11660-H07B), 1 *μ*g/ml and 10 *μ*g/ml monomeric Hsp72 IgG (mouse monoclonal IgG1, Abcam, ab47455), 1 *μ*g/ml and 10 *μ*g/ml of monomeric isotype IgG1 (Abcam, ab91353), 1 *μ*g/ml and 10 *μ*g/ml Hsp72-IgG complex (generated by incubating the Hsp72 and Hsp72 IgG1 antibody at a 1 : 3 ratio for 20 minutes prior to MDM treatment), 50 ng/ml LPS, and 1 *μ*g/ml IPF patient BALf IgG (with and without preincubation with 100 ng/ml Hsp72). Cell culture supernatant was retrieved after 24 hours of MDM cell treatment for analysis.

### 2.5. Immunohistochemistry

Immunohistochemistry for Hsp72 and C4d was performed on UIP and chronic HP lung biopsies. Hsp72 staining was performed by the SURF Immunohistochemistry using a BOND-MAX Automated Immunostainer at the QMRI, Little France, Edinburgh. C4d staining was performed using a BOND-MAX Automated Immunostainer by NHS Lothian at Edinburgh Royal Infirmary. Hsp72 staining used a peroxidase-DAB method, and C4d staining used an alkaline phosphatase staining method.

### 2.6. Statistical Analysis

Nonparametric statistical tests were used where indicated. Kaplan-Meier (KM) survival and a Cox proportional hazard regression analysis were used for patient survival analysis, and hazard ratios (HR) adjusted for patient clinical data were made.

## 3. Results

### 3.1. Total IgG Is Elevated in IPF Patient BALf but Decreased in the Serum Compared to Healthy Controls

**Figure 1:**
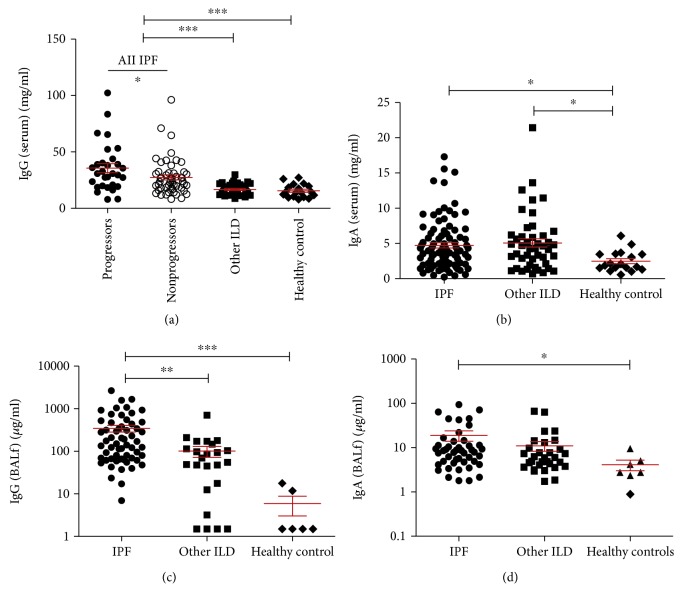
Total IgG and IgA concentrations in the serum and BALf were elevated in IPF. Total IgG was found to be significantly elevated in the serum of IPF patients compared to other ILD patients and healthy controls ((a) *p* = <0.05). IPF and other ILD patients had elevated IgA compared to healthy controls ((b) *p* = 0.016). BALf concentrations of IgG were elevated in IPF compared to other ILDs and healthy controls ((c) *p* = <0.001 and *p* = <0.0001, respectively; other ILDs to healthy controls not significant by Kruskal-Wallis). BALf concentrations of IgA were elevated in IPF compared to healthy controls ((d) *p* = 0.026; other ILDs to healthy controls not significant by Kruskal-Wallis). IPF progressors had elevated serum IgG compared to nonprogressors (*p* = 0.043). No differences were seen between IPF subgroups in serum IgA or BALf IgG or IgA. Statistical analysis between patient cohorts and healthy controls was performed using the Kruskal-Wallis test with Dunn's posttest. Comparison between IPF progressors and nonprogressors was analysed with the Mann-Whitney *U* test.

IPF is not considered a classical autoimmune disease; however, it has been reported that IPF patients express some features of classical autoimmunity such as elevated CXCL13 expression [8]. Increased titre of circulating IgG is a feature of classical autoimmune diseases such as systemic lupus erythematosus and rheumatoid arthritis [31, 32]. Therefore, we measured total IgG and IgA in the serum and BAL fluid (BALf) of a cohort of IPF patients; non-IPF ILD patients, henceforth referred to as “other ILD”; and healthy controls. IPF patients were found to have elevated IgG compared to both other ILD patients and healthy controls (Figure 1(a), *p* = <0.0001). Both IPF and other ILD patients had elevated serum IgA compared to healthy controls (Figure 1(b), *p* = 0.016). In the IPF cohort, patients can be split into “progressor” and “nonprogressor” subgroups, with IPF progression defined as ≥10% loss in VC or IPF-related death within 12 months of sampling. When the IPF cohort was split into these two subgroups, it was found that IPF progressors had an elevated concentration of serum IgG (*p* = 0.043). Conversely, in the BALf, elevated concentrations of IgG and IgA were found in IPF patients compared to healthy controls (Figures 1(c) and 1(d), IgG *p* = <0.0001, IgA *p* = 0.026). IPF patients were also found to have elevated IgG, but not IgA, compared to other ILDs. When comparing IPF patient subgroups, no differences were found in the BALf immunoglobulin concentrations.

### 3.2. The Distribution of Hsp72 Expression in IPF

**Figure 2:**
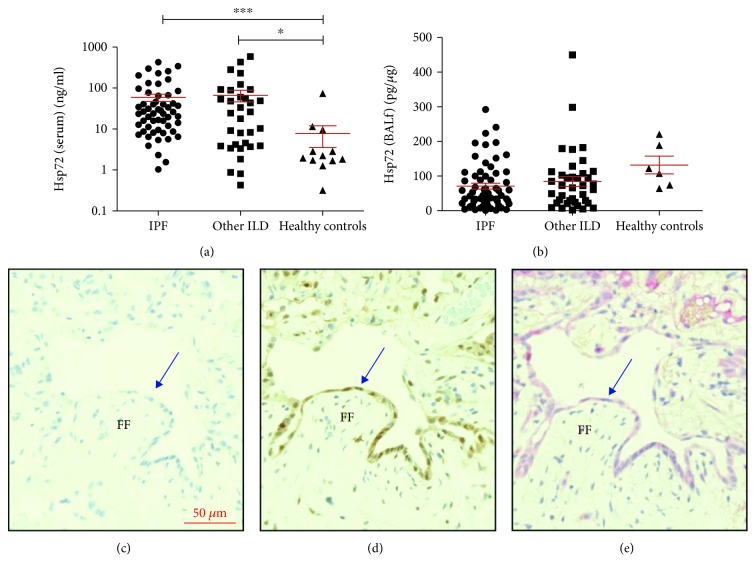
Elevated expression of Hsp72 in IPF patients occurred in lung epithelia and coincided with C4d deposition. IPF patient serum and BALf was measured for Hsp72 using a commercial ELISA (IPF *n* = 82, other ILDs *n* = 38, healthy controls *n* = 22). Serum concentrations of Hsp72 were elevated in ILD patients compared to healthy controls ((a) *p* = 0.002). Hsp72 concentrations in the BALf were normalised to total albumin content, and no difference was seen between ILD and healthy controls ((b) *p* = 0.059). Immunohistochemistry detection of Hsp72 and C4d was performed on 8 IPF biopsies with UIP ((c) unstained control, (d) Hsp72 detection, and (e) C4d detection). Detection of Hsp72 and C4d was seen in hyperplastic alveolar epithelia (denoted by blue arrows) near areas of fibrotic foci (FF). Statistics was performed using a Kruskal-Wallis test with Dunn's posttest.

Previous work has demonstrated the presence of anti-Hsp72 antibodies in IPF patients [14]; therefore, we measured concentrations of the target antigen in the serum and BALf of ILD patients and healthy controls (Figures 2(a) and 2(b)). Hsp72 was found to be elevated in the serum of IPF and other ILD patients compared to controls (Figure 2(a) *p* = 0.002), with no difference between ILD patient subgroups. Hsp72 concentrations measured in the BALf were not significantly different between ILD patients and healthy controls (Figure 2(b)).

Lung biopsies of 8 IPF patients showed high expression of Hsp72 in type II hyperplastic AECs near fibrotic foci (Figure 2(d)). Hsp72 was detectable in all samples but varied between patients. Hsp72 expression was also detected in bronchial cells, immune cells (macrophage and lymphoid cells), and the extracellular compartment.

C4d is a downstream product of the IgG-activated complement classical pathway and has previously been used as a marker of antibody-mediated rejection in transplants [33]. Therefore, detection of C4d deposition can be used to indicate the presence of IgG. We found a similar pattern of detection of C4d compared to Hsp72 in the IPF patient lung biopsies. This may therefore suggest the presence of anti-Hsp72 antibodies in IPF patient lungs.

### 3.3. Decreased BALf Anti-Hsp72 IgG and IgGAM Is Associated with Disease Progression

**Figure 3:**
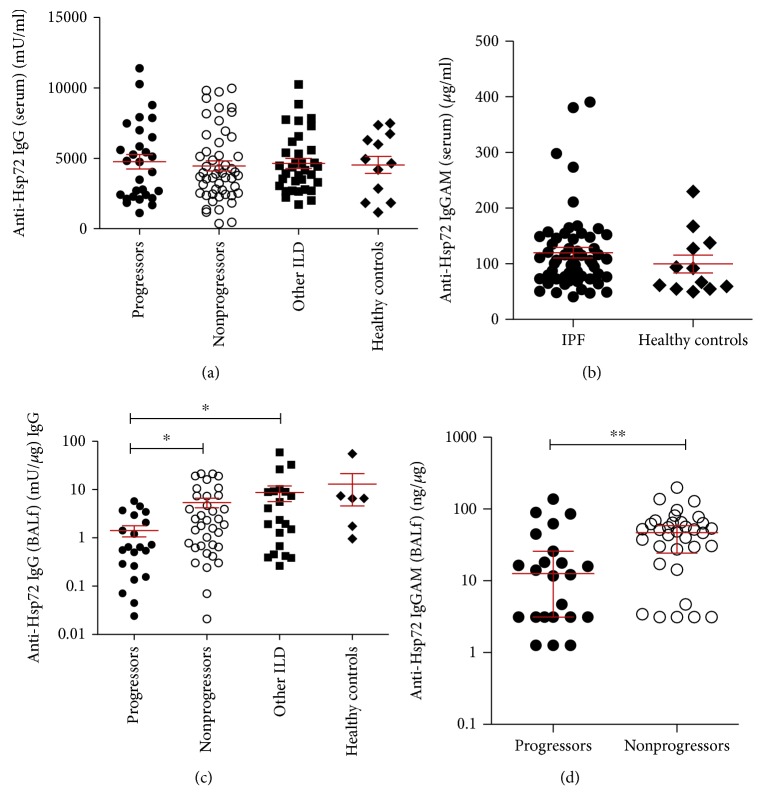
Anti-Hsp72 IgG and IgGAM concentrations are elevated in the BALf of IPF patient nonprogressors compared to progressors. Anti-Hsp72 IgG was measured in the serum and BALf using the in-house optimised anti-Hsp72 IgG ELISA and commercial anti-Hsp72 IgGAM ELISA (IPF *n* = 82, other ILDs *n* = 46, healthy controls *n* = 17). BALf concentrations of anti-Hsp72 IgG and IgGAM are standardised to BALf total IgG. Between ILD subgroups and healthy controls, no significant difference was seen in the serum anti-Hsp72 IgG ((a) *p* = 0.72) or anti-Hsp72 IgGAM ((b) *p* = 0.24). No significant difference was seen between IPF progressor and nonprogressor patient serum anti-Hsp72 IgG ((a) *p* = 0.83) or serum anti-Hsp72 IgGAM ((b) *p* = 0.95) concentrations. Elevated concentrations of BALf anti-Hsp72 IgG ((c) *p* = 0.014) and anti-Hsp72 IgGAM ((d) *p* = 0.005) were seen in IPF nonprogressors compared to progressors. Statistical analysis was performed using Kruskal-Wallis with Dunn's posttest and Mann-Whitney *U* tests.

To test whether increased anti-Hsp72 antibody expression could be a determining factor in IPF progression, we measured anti-Hsp72 IgG concentrations in the serum and BALf using both an in-house optimised ELISA and a commercially available anti-Hsp72 IgGAM ELISA (Figure 3). No significant difference was observed between IPF patients, other ILD patients, and healthy controls in anti-Hsp72 IgG serum concentrations (Figure 3(a), *p* = 0.72) or anti-Hsp72 IgGAM concentrations (Figure 3(b), *p* = 0.24). There was no difference in anti-Hsp72 IgG or IgGAM serum concentrations when patients were split into progressors and nonprogressors (Figures 3(a) and 3(b)).

No significant difference was observed in BALf anti-Hsp72 IgG between IPF and other ILD patients (Figure 3(c), *p* = 0.084). Anti-Hsp72 IgGAM was not measured in other ILD patient serum and BALf. We observed significantly lower concentrations of anti-Hsp72 IgG (Figure 3(c), *p* = 0.014) and anti-Hsp72 IgGAM (Figure 3(d), *p* = 0.005) in IPF progressors compared to nonprogressors.

### 3.4. Increased Concentrations of BALf Anti-Hsp72 IgGAM, but Not Anti-Hsp72 IgG, Are Associated with Increased Patient Survival

Antibody concentrations did not associate with disease severity (as measured by patient percentage predicted VC and TL_CO_ values, supplementary Figure 1). However, anti-Hsp72 antibodies in the BALf were elevated IPF patients who were nonprogressors compared to progressors. We analysed the association between BALf anti-Hsp72 antibody (and total IgG and IgA) concentrations and patient survival.

**Figure 4:**
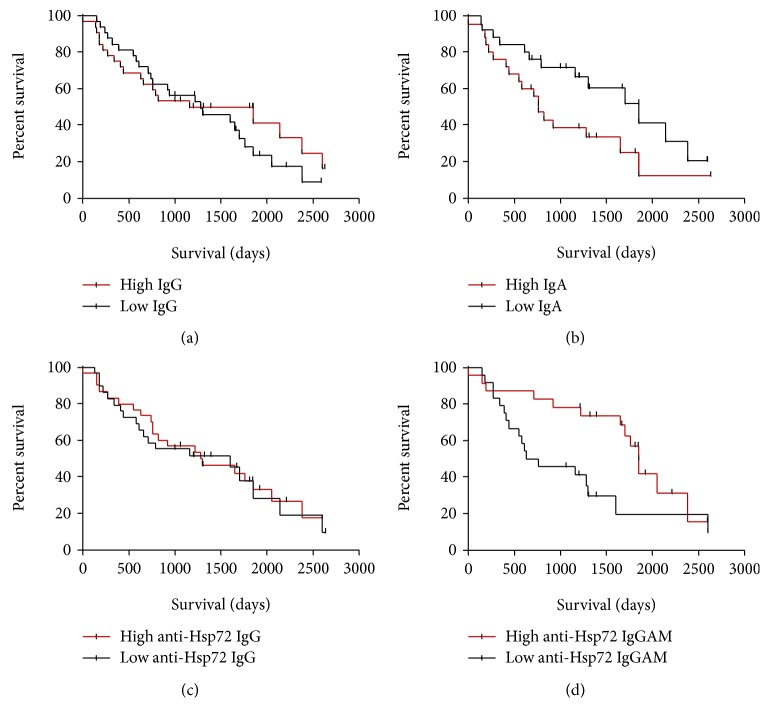
IPF patients with raised concentrations of BALf anti-Hsp72 IgGAM have longer survival rates. Total BALf IgG, IgA and anti-Hsp72 IgG IgGAM were split between high (red lines) and low (black lines) expression rates by the median value. A Kaplan-Meier survival curve from the date of the BAL sample was made to either death or the census date. No association with survival was seen in total IgG ((a) HR 0.82, 95% CI 0.45-1.49, *p* = 0.51) or anti-Hsp72 IgG ((c) HR 1.38, 95% CI 0.50-1.74, *p* = 0.33). Total IgA ((b) HR 2.09, 95% CI 1.01-4.32, *p* = 0.048) and anti-Hsp72 IgGAM ((d) HR 0.44, 95% CI 0.20-0.92, *p* = 0.018) associated with improved patient survival. Total IgA and anti-Hsp72 IgGAM were reanalysed using a Cox proportional hazard model adjusting for age, sex, smoking, and percentage predicted VC and T_L_CO. After adjustment, total IgA showed no association with patient survival (adjusted HR 0.85, *p* = 0.77), whilst elevated anti-Hsp72 IgGAM did show a significant association with survival (adjusted HR 0.62, 95% CI 0.45-0.85, *p* = 0.003).

Patients were dichotomised into high expressing and low expressing patients according to the median value to generate a Kaplan-Meier (KM) survival curve (Figure 4). In the unadjusted model, lower concentrations of IgA but higher concentrations of anti-Hsp72 IgGAM were found to associate with increased patient survival (Figure 4; IgA HR = 2.085, *p* = 0.048; anti-Hsp72 IgGAM HR = 0.44, *p* = 0.018).

Using a Cox proportional hazard model, and after adjusting for age, sex, smoking status, percentage-predicted VC, and percentage-predicted T_L_CO, no significant association was identified for total BALf IgA and survival but increased BALf anti-Hsp72 IgGAM was associated with improved patient survival (adjusted HR 0.62, 95% CI 0.45-0.85; *p* = 0.003).

### 3.5. MDM Response to Hsp72-IgG Complexes

**Figure 5:**
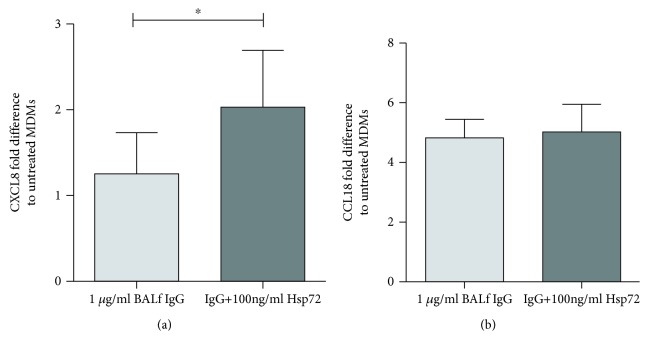
Hsp72-IgG complexes induce CXCL8 secretion from *in vitro* MDM culture. MDM cell culture supernatant was analysed for CXCL8 (a) and CCL18 (b) by ELISA after treatment with 1 *μ*g/ml IgG isolated from BALf of IPF patients ± 100 ng/ml Hsp72 (IPF patient *n* = 6, repeated twice) for 24 hours. 1 *μ*g/ml of BALf IgG did not lead to any overt fold change in CXCL8 secretion in comparison to untreated cells, but preincubation of IgG with Hsp72 led to increased CXCL8 secretion ((a), *p* = 0.031). A 4-fold increase in MDM CCL18 secretion was seen in response to BALf IgG, with Hsp72 preincubation having no effect ((b), *p* = 0.84). Statistical tests to compare BALf IgG with preincubated BALf IgG used a Wilcoxon test; no statistical comparison was made with untreated cells.

Hypothesising that increased survival through anti-Hsp72 antibodies is mediated by immune cells, we isolated IgG from the BALf of IPF patients and cultured the IgG on MDMs with or without prior coincubation of IgG with Hsp72 (Figure 5). MDMs secreted CXCL8 when induced with IPF patient BALf IgG coincubated with 100 ng/ml Hsp72 (Figure 5(a), *p* = 0.031). CCL18 secretion had no changes when cultured with IPF patient BALf IgG with and without Hsp72; under both conditions, there was a 4-fold increase in CCL18 secretion compared to untreated cells (Figure 5(b)).

## 4. Discussion

The role of autoimmunity and specifically immunoglobulins, in the pathogenesis of IPF, is unclear. Our initial observations focussed on total immunoglobulin concentrations as increased titres of circulating IgG are normally reported in classical autoimmune diseases such as systemic lupus erythematosus and rheumatoid arthritis [31, 32]. In this study, we found that BALf IgG (and IgM, supplementary Figure 3) levels were significantly elevated in IPF patients compared with other ILDs, which in turn were higher than controls (significantly higher with respect to IgG and a trend seen with IgA and IgM). BALf IgA was seen to be significantly elevated in IPF with respect to controls but comparable to other ILDs. In the serum, only IgG, but not IgA or IgM, levels were higher in IPF versus other ILD patients. These data suggest a heightened humoral response in the lungs of IPF patients compared to other ILD patients, which is consistent with previous observations of organised B cells in IPF lungs [9, 10]. Elevated serum IgG can be associated with autoimmune disorders such as rheumatoid arthritis, and so, this IgG response in IPF could be due to the loss of self-tolerance seen in IPF [12–15]. Indeed, the presence of various autoantibodies supports this hypothesis [14, 18–23].

Having found supporting evidence for a potential autoimmune process in IPF, we sought to investigate anti-Hsp72 antibodies in our patients. Various autoantibodies have been described in IPF [18–23], but our group has in the past described putative Hsp72 autoantigen expression in IPF [16, 17], and others have shown circulating anti-Hsp72 antibody to be associated with poor outcome in IPF [14] We therefore initially hypothesised that anti-Hsp72 IgG would be elevated in the serum and BAL and associate with disease progression and poorer survival. However, we found that serum anti-Hsp72 antibodies were similar in the IPF progressor versus nonprogressors and indeed no different to patients with other ILDs and controls. These results seem to contradict previous findings [14]. However, a crucial methodological difference in the detection of anti-Hsp72 IgG may be responsible for this discrepancy; we assayed anti-Hsp72 IgG targeted to rHsp72 in native form, and Kahloon et al. found that detection using nondenatured rHsp72 had no associations to clinical outcomes [14]. Furthermore, and contrary to our hypothesis, we found higher levels of anti-Hsp72 IgG and IgGAM in the BALf in IPF nonprogressors compared to progressors, and for Hsp72 IgGAM, higher BALf expression was associated with increased survival in an adjusted multivariate analysis model.

Given these data, we speculate that Hsp72 antibodies are natural in origin in IPF. Hsp72 fits the archetypal role of a natural autoantibody antigen. Natural autoantibodies are hypothesised to mediate immune responses typically, though not exclusively through autoreactive IgM, with anti-leukocyte IgM regulating innate and adaptive immune mechanisms (reviewed by Lobo et al. [34]). It has been observed that the presence of anti-dsDNA IgM may reduce the severity of lupus nephritis in SLE patients [35]. However, in our studies, total IgM did not associate with ILD disease or with IPF patient survival (Supplementary Figure 3). Natural autoreactive IgG has also been demonstrated to have a mediating role in inflammation in other disease models [36, 37]. We observed no association of total BALf immunoglobulin with IPF outcomes; however, the presence of various antibodies in IPF including Hsp72 [18–22, 38] supports the possibility of antigen-specific natural antibodies modulating disease pathogenesis and impacting patient outcomes.

The provenance of anti-Hsp72 antibodies in the lungs of IPF patients is uncertain. We speculate that anti-Hsp72 immunoglobulins in the lung are natural, as they are detectable in healthy controls, but we cannot rule out that the pulmonary antibodies in particular do not arise until after disease onset. The establishment of organised and proliferative B cell germinal centers in IPF lungs [9, 10] and the elevation of immunoglobulins seen in the BALf in this study support a reactive process. It may be possible that the lung expression of anti-Hsp72 IgG arises due to the impairment of T_Reg_ lymphocytes [13]. The presence of serum anti-Hsp72 IgG may be due to leakage from the diseased lung, but no correlation between the serum and BALf IgG or anti-Hsp72 IgG was seen (data not shown).

Macrophages are reportedly activated in IPF [10, 39, 40], and as they express a suite of Fc*γ* receptors, it seems highly likely they would be cardinal cells to respond to a functional antibody in IPF. We found that MDMs treated with IgG isolated from patient BALf secreted more CXCL8 when exposed to Hsp72 than those cells treated with the IgG alone. This was also true using commercial anti-Hsp72-derived antibodies (supplementary Figure 4). Macrophages have previously been suggested to be a major source of CCL18 in IPF [40], which our data also supports and shows this may be due to IgG stimulation of macrophages. Polymorphisms in Fc*γ*RIIa and Fc*γ*RIIIb in IPF patients may alter macrophage responses [41, 42]. Further work is required to fully determine the repertoire of antibodies in IPF and their functional relevance to macrophages and other Fc*γ*R expressing cells, such as neutrophils.

The focus of autoimmunity in IPF, often targeting epithelial antigens, has conventionally been hypothesised as being pathological in nature. Here, we report that elevated serum IgG is a feature of IPF, in keeping with other classical autoimmune diseases. However, the intrapulmonary autoantibody to Hsp72 may have a protective role. We speculate that anti-Hsp72 IgG is natural in origin and promotes a homeostatic immune response.

## Figures and Tables

**Table 1:** Patient demographics.

	All IPF patients (*n* = 107)^∗^	IPF progressors (*n* = 30)	IPF nonprogressors (*n* = 52)	Other ILDs (*n* = 66)	Healthy controls (*n* = 19)
Sex (% male)	73.8	83.3	67.3	57.6	42.1
Median age at BAL (+range)	73 (51-87)	71.5 (51-85)	73 (57-85)	64 (41-81)	66 (44-70)
BAL tested (%)	63.6	76.7	71.1	56	36.8
BAL baseline % predicted vital capacity (VC) mean ± SEM	89.7 ± 2.7	83.5 ± 4.5	92.4 ± 3.3	88.7 ± 3.1	99.3 ± 5.3
BAL baseline % predicted T_L_CO mean ± SEM	53.8 ± 1.8	51.0 ± 2.7	55.5 ± 2.3	57.0 ± 2.4	n/a
Median age at serum (+range)	74 (51-89)	74 (51-89)	74 (52-87)	65 (31-85)	66 (44-87)
Serum tested (%)	76.6	93.3	94.2	78.8	79.0
Serum baseline % predicted vital capacity (VC) mean ± SEM	86.4 ± 2.4	79.7 ± 3.7	90.2 ± 3.1	87.1 ± 2.8	99.3 ± 5.3
Serum baseline % predicted T_L_CO mean ± SEM	53.4 ± 1.8	46.1 ± 3.4	49.1 ± 1.9	57.6 ± 2.2	n/a

^∗^At the time of analysis, 25 of the 107 IPF subjects had not been followed up for at least 12 months; hence, there are 30 progressors, 52 nonprogressors, and 25 not determined at the time of analysis.

## Data Availability

The individual patient data used to support the findings of this study are restricted by the NHS Lothian Research ethics board in order to protect patient privacy. Relevant data is included within the article.

## Supplementary Materials

Supplementary 1 Supplementary Table 1: shows the breakdown of the 3 groups of patients who constituted the “other ILD” patient group used for analysis in this study.

Supplementary 2 Supplementary Figure 1: the concentrations of serum and BALf anti-Hsp72 IgG and IgGAM did not correlate with decreased percentage predicted values of VC or T_L_CO. BALf anti-Hsp72 antibody concentrations are not a marker of disease severity in IPF. BALf anti-Hsp72 IgG (a, b) and IgGAM (c, d) concentrations were correlated against the percentage predictive values of VC and T_L_CO in IPF patients. Correlations were performed using a Spearman rank. BALf Anti-Hsp72 IgG concentrations were standardised to BALf total IgG concentrations. BALf anti-Hsp72 IgG had no correlation with VC ((a) *r* = 0.083, *p* = 0.54) or T_L_CO ((b) *r* = −0.058, *p* = 0.69). BALf anti-Hsp72 IgGAM had no correlation to VC ((c) *r* = 0.11, *p* = 0.40) or T_L_CO ((d) *r* = 0.13, *p* = 0.35).

Supplementary 3 Supplementary Figure 2: BALf anti-Hsp72 IgG normalised to total protein is elevated in IPF compared to healthy controls. BALf anti-Hsp72 IgG was normalised to total protein concentrations measured by BCA. IPF progressors (*p* = <0.001) and nonprogressors (*p* = <0.0001) were elevated compared to healthy controls, and nonprogressors had elevated concentrations compared to other ILDs (*p* = <0.0001). A nonsignificant elevation in anti-Hsp72 IgG was seen in nonprogressors compared to progressors (*p* = 0.096).

Supplementary 4 Supplementary Figure 3: serum IgM had no difference between all ILD patients and healthy controls (*p* = 0.30). BALf IgM was significantly elevated in IPF patients compared to other ILD patients (*p* = 0.003). Serum ((b) HR = 1.03, 95% CI 0.58-1.82) and BALf ((d) HR 0.99, 95% CI 0.49-2.02) IgM levels were not associated with IPF patient outcomes or survival.

Supplementary 5 Supplementary Figure 4: Hsp72-IgG complexes induce CXCL8 secretion from *in vitro* MDM culture. MDM cell culture supernatant was analysed for CXCL8 (a, c) and CCL18 (b, d) by ELISA after treatment with 100 ng/ml Hsp72, 1 *μ*g/ml anti-Hsp72 IgG, 1 *μ*g/ml Hsp72-IgG complex, or 50 ng/ml LPS ((a, b) *n* = 7) for 24 hours. Elevated CXCL8 secretion was seen in response to 10 *μ*g/ml mouse monomeric Hsp72 IgG and Hsp72-IgG complexes ((a) *p* = <0.05 and *p* = <0.001, respectively). No secretion of CCL18 was seen in response to mouse anti-Hps72 IgG ((b) *p* = 0.24, LPS excluded). (c) and (d) show the measured concentrations of CXCL8 and CCL18 after MDM culture with 1 *μ*g/ml isolated BALf IgG with and without preculture with 100 ng/ml Hsp72. A significant difference was observed in CXCL8 ((c) *p* = 0.012) and CCL18 ((d) *p* = 0.032) secretion; however, poststats failed to identify the difference which is likely due to the experiment being underpowered. CXCL8 secretion was induced by BALf IgG preincubated with 100 ng/ml Hsp72 in a nonsignificant trend compared to BALf IgG alone ((c) *p* = 0.063).

Supplementary 6 Supplementary Figure 5: the in-house optimised and commercial ELISA had a correlation in anti-Hsp72 antibody detection in the BALf. Serum concentrations of anti-Hsp72 antibodies did not correlate between the two ELISAs ((a) *p* = 0.24, Spearman *r* = 0.16). BALf concentrations were used normalised to total IgG as nonnormalised data for anti-Hsp72 IgGAM is unavailable. BALf concentrations of anti-Hsp72 antibodies correlated between ELISAs ((b) *p* = 0.0009, Spearman *r* = 0.48). Anti-Hsp72 IgG concentrations not normalised to total IgG had a near significant correlation with total IgG normalised anti-Hsp72 IgGAM concentrations (not shown, *p* = 0.051, Spearman *r* = 0.30).

## References

[1] Martinez F. J., Collard H. R., Pardo A. (2017). Idiopathic pulmonary fibrosis. *Nature Reviews Disease Primers*.

[2] King T. E., Tooze J. A., Schwarz M. I., Brown K. R., Cherniack R. M. (2001). Predicting survival in idiopathic pulmonary fibrosis: scoring system and survival model. *American Journal of Respiratory and Critical Care Medicine*.

[3] Flaherty K. R., King T. E., Raghu G. (2004). Idiopathic interstitial pneumonia: what is the effect of a multidisciplinary approach to diagnosis?. *American Journal of Respiratory and Critical Care Medicine*.

[4] Ryerson C. J., Urbania T. H., Richeldi L. (2013). Prevalence and prognosis of unclassifiable interstitial lung disease. *European Respiratory Journal*.

[5] King T. E., Pardo A., Selman M. (2011). Idiopathic pulmonary fibrosis. *The Lancet*.

[6] Nagaya H., Sieker H. O. (1972). Pathogenetic mechanisms of interstitial pulmonary fibrosis in patients with serum antinuclear factor. A histologic and clinical correlation. *The American Journal of Medicine*.

[7] Dall’Aglio P. P., Pesci A., Bertorelli G., Brianti E., Scarpa S. (1988). Study of immune complexes in bronchoalveolar lavage fluids. *Respiration*.

[8] Vuga L. J., Tedrow J. R., Pandit K. V. (2014). C-X-C motif chemokine 13 (CXCL13) is a prognostic biomarker of idiopathic pulmonary fibrosis. *American Journal of Respiratory and Critical Care Medicine*.

[9] Wallace W. A. H., Howie S. E. M., Krajewski A. S., Lamb D. (1996). The immunological architecture of B-lymphocyte aggregates in cryptogenic fibrosing alveolitis. *Journal of Pathology*.

[10] Marchal-Sommé J., Uzunhan Y., Marchand-Adam S. (2006). Cutting edge: nonproliferating mature immune cells form a novel type of organized lymphoid structure in idiopathic pulmonary fibrosis. *Journal of Immunology*.

[11] Donahoe M., Valentine V. G., Chien N. (2015). Autoantibody-targeted treatments for acute exacerbations of idiopathic pulmonary fibrosis. *PLoS One*.

[12] Gilani S. R., Vuga L. J., Lindell K. O. (2010). CD28 down-regulation on circulating CD4 T-cells is associated with poor prognoses of patients with idiopathic pulmonary fibrosis. *PLoS One*.

[13] Kotsianidis I., Nakou E., Bouchliou I. (2009). Global impairment of CD4^+^CD25^+^FOXP3^+^ regulatory T cells in idiopathic pulmonary fibrosis. *American Journal of Respiratory and Critical Care Medicine*.

[14] Kahloon R. A., Xue J., Bhargava A. (2013). Patients with idiopathic pulmonary fibrosis with antibodies to heat shock protein 70 have poor prognoses. *American Journal of Respiratory and Critical Care Medicine*.

[15] Feghali-Bostwick C. A., Tsai C. G., Valentine V. G. (2007). Cellular and humoral autoreactivity in idiopathic pulmonary fibrosis. *Journal of Immunology*.

[16] Wallace W. A., Roberts S. N., Caldwell H. (1994). Circulating antibodies to lung protein(s) in patients with cryptogenic fibrosing alveolitis. *Thorax*.

[17] Wallace W. A., Schofield J. A., Lamb D., Howie S. E. (1994). Localisation of a pulmonary autoantigen in cryptogenic fibrosing alveolitis. *Thorax*.

[18] Kurosu K., Takiguchi Y., Okada O. (2008). Identification of annexin 1 as a novel autoantigen in acute exacerbation of idiopathic pulmonary fibrosis. *Journal of Immunology*.

[19] Fujita J., Dobashi N., Ohtsuki Y. (1999). Elevation of anti-cytokeratin 19 antibody in sera of the patients with idiopathic pulmonary fibrosis and pulmonary fibrosis associated with collagen vascular disorders. *Lung*.

[20] Dobashi N., Fujita J., Ohtsuki Y. (1998). Detection of anti-cytokeratin 8 antibody in the serum of patients with cryptogenic fibrosing alveolitis and pulmonary fibrosis associated with collagen vascular disorders. *Thorax*.

[21] Taillé C., Grootenboer-Mignot S., Boursier C. (2011). Identification of periplakin as a new target for autoreactivity in idiopathic pulmonary fibrosis. *American Journal of Respiratory and Critical Care Medicine*.

[22] Yang Y., Fujita J., Bandoh S. (2002). Detection of antivimentin antibody in sera of patients with idiopathic pulmonary fibrosis and non-specific interstitial pneumonia. *Clinical and Experimental Immunology*.

[23] Shum A. K., Alimohammadi M., Tan C. L. (2013). BPIFB1 is a lung-specific autoantigen associated with interstitial lung disease. *Science Translational Medicine*.

[24] Feghali-Bostwick C. A., Wilkes D. S. (2011). Autoimmunity in idiopathic pulmonary fibrosis: are circulating autoantibodies pathogenic or epiphenomena?. *American Journal of Respiratory and Critical Care Medicine*.

[25] Nagele E. P., Han M., Acharya N. K., DeMarshall C., Kosciuk M. C., Nagele R. G. (2013). Natural IgG autoantibodies are abundant and ubiquitous in human sera, and their number is influenced by age, gender, and disease. *PLoS One*.

[26] Quan C. P., Berneman A., Pires R., Avrameas S., Bouvet J. P. (1997). Natural polyreactive secretory immunoglobulin A autoantibodies as a possible barrier to infection in humans. *Infection and Immunity*.

[27] Seigneurin J. M., Guilbert B., Bourgeat M. J., Avrameas S. (1988). Polyspecific natural antibodies and autoantibodies secreted by human lymphocytes immortalized with Epstein-Barr virus. *Blood*.

[28] Daffa N. I., Tighe P. J., Corne J. M., Fairclough L. C., Todd I. (2015). Natural and disease-specific autoantibodies in chronic obstructive pulmonary disease. *Clinical and Experimental Immunology*.

[29] Muro Y., Sugiura K., Shiraki A., Ishii N., Hashimoto T., Akiyama M. (2014). Detection of autoantibodies to periplakin and envoplakin in paraneoplastic pemphigus but not idiopathic pulmonary fibrosis using full-length recombinant proteins. *Clinica Chimica Acta*.

[30] Travis W. D., King T. E., Bateman E. D. (2002). American Thoracic Society/European Respiratory Society international multidisciplinary consensus classification of the idiopathic interstitial pneumonias. *American Journal of Respiratory and Critical Care Medicine*.

[31] Upton J. (2015). Immunodeficiencies with hypergammaglobulinemia: a review. *LymphoSign Journal*.

[32] Boes M., Schmidt T., Linkemann K., Beaudette B. C., Marshak-Rothstein A., Chen J. (2000). Accelerated development of IgG autoantibodies and autoimmune disease in the absence of secreted IgM. *Proceedings of the National Academy of Sciences of the United States of America*.

[33] Murata K., Baldwin Iii W. M. (2009). Mechanisms of complement activation, C4d deposition, and their contribution to the pathogenesis of antibody-mediated rejection. *Transplantation Reviews*.

[34] Lobo P. I., Brayman K. L., Okusa M. D. (2014). Natural IgM anti-leucocyte autoantibodies (IgM-ALA) regulate inflammation induced by innate and adaptive immune mechanisms. *Journal of Clinical Immunology*.

[35] Villalta D., Bizzaro N., Bassi N. (2013). Anti-dsDNA antibody isotypes in systemic lupus erythematosus: IgA in addition to IgG anti-dsDNA help to identify glomerulonephritis and active disease. *PLoS One*.

[36] Quintana F. J., Hagedorn P. H., Elizur G., Merbl Y., Domany E., Cohen I. R. (2004). Functional immunomics: microarray analysis of IgG autoantibody repertoires predicts the future response of mice to induced diabetes. *Proceedings of the National Academy of Sciences of the United States of America*.

[37] Mannoor K., Matejuk A., Xu Y., Beardall M., Chen C. (2012). Expression of natural autoantibodies in MRL-lpr mice protects from lupus nephritis and improves survival. *Journal of Immunology*.

[38] Fahim A., Chong M. C., Crooks M. G., Hart S. P. (2012). Idiopathic pulmonary fibrosis is associated with circulating antiepithelial antibodies. *Lung*.

[39] Prasse A., Pechkovsky D. V., Toews G. B. (2006). A vicious circle of alveolar macrophages and fibroblasts perpetuates pulmonary fibrosis via CCL18. *American Journal of Respiratory and Critical Care Medicine*.

[40] Pechkovsky D. V., Prasse A., Kollert F. (2010). Alternatively activated alveolar macrophages in pulmonary fibrosis-mediator production and intracellular signal transduction. *Clinical Immunology*.

[41] Bournazos S., Bournazou I., Murchison J. T. (2010). Fc*γ* receptor IIIb (CD16b) polymorphisms are associated with susceptibility to idiopathic pulmonary fibrosis. *Lung*.

[42] Bournazos S., Grinfeld J., Alexander K. M. (2010). Association of Fc*γ*RIIa R131H polymorphism with idiopathic pulmonary fibrosis severity and progression. *BMC Pulmonary Medicine*.

