# Supplementary material for: Intrapulmonary Autoantibodies to HSP72 Are Associated with Improved Outcomes in IPF
Source: J Immunol Res. 2019 Apr 11;2019:1845128. doi: 10.1155/2019/1845128 (PMC6487088; doi:10.1155/2019/1845128)
Supplement: Supplementary 1 — Supplementary Table 1: shows the breakdown of the 3 groups of patients who constituted the “other ILD” patient group used for analysis in this study. [file 1845128.f1.docx]

## Figures/Tables

Table 1 Patient Demographics

|  | All IPF patients (n=107)* | IPF progressors (n=30) | IPF non-progressors (n=52) | Other ILDs (n=66) | Healthy controls (n=19) |
| --- | --- | --- | --- | --- | --- |
| Sex (% male) | 73.8 | 83.3 | 67.3 | 57.6 | 42.1 |
| Median Age at BAL (+range) | 73 (51-87) | 71.5 (51-85) | 73 (57-85) | 64 (41-81) | 66 (44-70) |
| BAL tested (%) | 63.6 | 76.7 | 71.1 | 56 | 36.8 |
| BAL Baseline % Predicted Vital Capacity (VC) Mean ±SEM | 89.7 ±2.7 | 83.5 ±4.5 | 92.4 ±3.3 | 88.7 ±3.1 | 99.3 ±5.3 |
| BAL Baseline % Predicted T_L_CO Mean ±SEM | 53.8 ±1.8 | 51.0 ±2.7 | 55.5 ±2.3 | 57.0 ±2.4 | n/a |
| Median Age at serum (+range) | 74 (51-89) | 74 (51-89) | 74 (52-87) | 65 (31-85) | 66 (44-87) |
| Serum tested (%) | 76.6 | 93.3 | 94.2 | 78.8 | 79.0 |
| Serum Baseline % Predicted Vital Capacity (VC) Mean ±SEM | 86.4 ±2.4 | 79.7 ±3.7 | 90.2 ±3.1 | 87.1 ±2.8 | 99.3 ±5.3 |
| Serum Baseline % Predicted T_L_CO Mean ±SEM | 53.4 ±1.8 | 46.1 ±3.4 | 49.1 ±1.9 | 57.6 ±2.2 | n/a |

**At time of analysis 25 of the 107 IPF subjects had not been followed up for at least 12 months, hence there are 30 progressors, 52 non-progressors and 25 not determined at time of analysis.*
