# Supplementary material for: Intrapulmonary Autoantibodies to HSP72 Are Associated with Improved Outcomes in IPF
Source: J Immunol Res. 2019 Apr 11;2019:1845128. doi: 10.1155/2019/1845128 (PMC6487088; doi:10.1155/2019/1845128)
Supplement: Supplementary 2 — Supplementary Figure 1: the concentrations of serum and BALf anti-Hsp72 IgG and IgGAM did not correlate with decreased percentage predicted values of VC or TLCO. BALf anti-Hsp72 antibody concentrations are not a marker of disease severity in IPF. BALf anti-Hsp72 IgG (a, b) and IgGAM (c, d) concentrations were correlated against the percentage predictive values of VC and TLCO in IPF patients. Correlations were performed using a Spearman rank. BALf Anti-Hsp72 IgG concentrations were standardised to BALf total IgG concentrations. BALf anti-Hsp72 IgG had no correlation with VC ((a) r = 0.083, p = 0.54) or TLCO ((b) r = −0.058, p = 0.69). BALf anti-Hsp72 IgGAM had no correlation to VC ((c) r = 0.11, p = 0.40) or TLCO ((d) r = 0.13, p = 0.35). [file 1845128.f2.docx]

Anti-Hsp72 antibodies do not associate with lung function. The association in IPF patients between disease severity (percentage predicted VC and TL_CO_) and serum/BALf anti-Hsp72 antibody concentrations was analysed by Spearman’s rank. From this analysis there was no evident correlation between serum or BALf anti-Hsp72 antibody concentrations and lung function from the point when the sample was taken. This possibly suggests that anti-Hsp72 antibodies do not have an impact on the initial lung function decline seen in IPF patients.


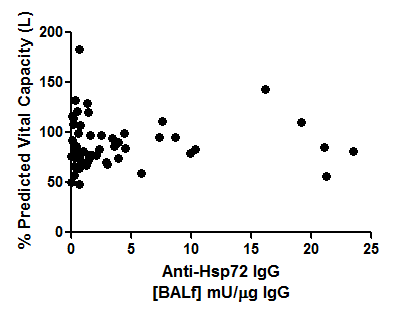

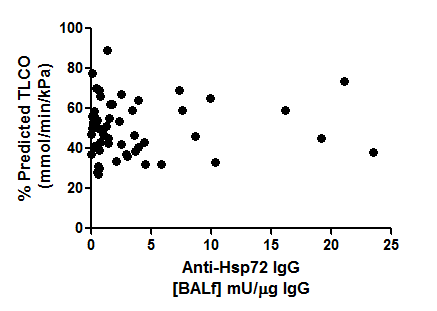

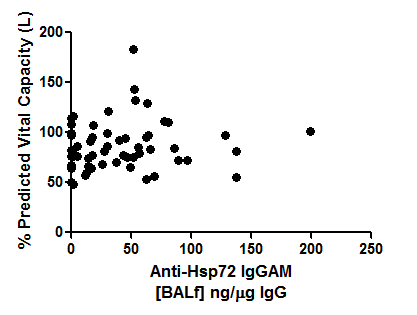

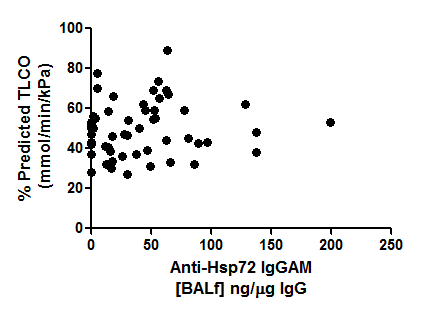


A

B

C

D

Supplementary figure 1. The concentrations of serum and BALf anti-Hsp72 IgG and IgGAM did not correlate with decreased percentage predicted values of VC or T_L_CO. BALf anti-Hsp72 antibody concentrations are not a marker of disease severity in IPF. BALf anti-Hsp72 IgG (A and B) and IgGAM (C and D) concentrations were correlated against the percentage predictive values of VC and T_L_CO in IPF patients. Correlations were performed using a Spearman rank. BALf Anti-Hsp72 IgG concentrations were standardised to BALf total IgG concentrations. BALf anti-Hsp72 IgG had no correlation with VC (A, r=0.083, p=0.54) or T_L_CO (B, r=-0.058, p=0.69). BALf anti-Hsp72 IgGAM had no correlation to VC (C, r=0.11, p=0.40) or T_L_CO (D, r=0.13, p=0.35).
