# Supplementary material for: Intrapulmonary Autoantibodies to HSP72 Are Associated with Improved Outcomes in IPF
Source: J Immunol Res. 2019 Apr 11;2019:1845128. doi: 10.1155/2019/1845128 (PMC6487088; doi:10.1155/2019/1845128)
Supplement: Supplementary 3 — Supplementary Figure 2: BALf anti-Hsp72 IgG normalised to total protein is elevated in IPF compared to healthy controls. BALf anti-Hsp72 IgG was normalised to total protein concentrations measured by BCA. IPF progressors (p = <0.001) and nonprogressors (p = <0.0001) were elevated compared to healthy controls, and nonprogressors had elevated concentrations compared to other ILDs (p = <0.0001). A nonsignificant elevation in anti-Hsp72 IgG was seen in nonprogressors compared to progressors (p = 0.096). [file 1845128.f3.docx]

BALf anti-Hsp72 IgG normalised to total protein is elevated compared to healthy controls. As an alternative to total IgG as a normalisation strategy total protein was used as a comparison.


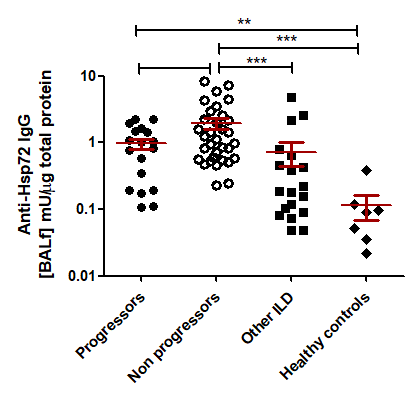


p=0.096

Supplementary figure 2: BALf anti-Hsp72 IgG normalised to total protein is elevated in IPF compared to healthy controls. BALf anti-Hsp72 IgG was normalised to total protein concentrations measured by BCA. IPF progressors (p=<0.001) and non progressors (p=<0.0001) were elevated compared to healthy controls, and non-progressors had elevated concentrations compared to Other ILDs (p=<0.0001). A non-significant elevation in anti-Hsp72 IgG was seen in non-progressors compared to progressors (p=0.096).
