# Supplementary material for: Intrapulmonary Autoantibodies to HSP72 Are Associated with Improved Outcomes in IPF
Source: J Immunol Res. 2019 Apr 11;2019:1845128. doi: 10.1155/2019/1845128 (PMC6487088; doi:10.1155/2019/1845128)
Supplement: Supplementary 4 — Supplementary Figure 3: serum IgM had no difference between all ILD patients and healthy controls (p = 0.30). BALf IgM was significantly elevated in IPF patients compared to other ILD patients (p = 0.003). Serum ((b) HR = 1.03, 95% CI 0.58-1.82) and BALf ((d) HR 0.99, 95% CI 0.49-2.02) IgM levels were not associated with IPF patient outcomes or survival. [file 1845128.f4.docx]

Total IgM concentrations were elevated in the BALf of IPF patients but did not associate with disease outcomes. Total IgM was measured in the serum and BALf of our cohort. In the serum no significant difference was seen between ILD and non-ILD conditions, further more total IgM did not associate with IPF subgroups (progressors and non progressors), or with IPF patient survival. In the BALf, total IgM was elevated in IPF patients compared to other ILD patients, however there was no association with IPF patient subgroups or survival. In conclusion, this data may support an ongoing local humoral response in IPF pathogenesis greater than in other ILD patients, but the impact of total IgM in disease outcome is of little impact on lung function or patient survival.

A


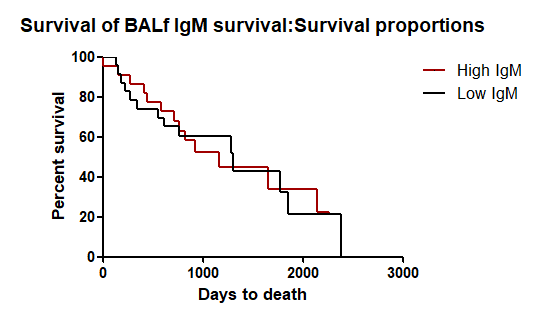

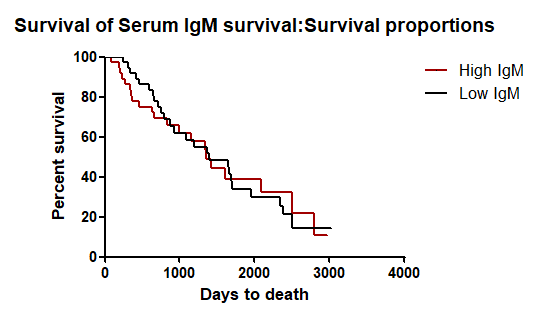


HR= 1.019

HR= 0.9731


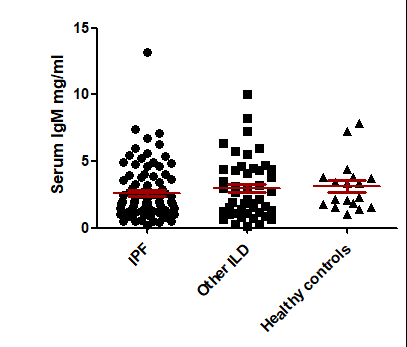

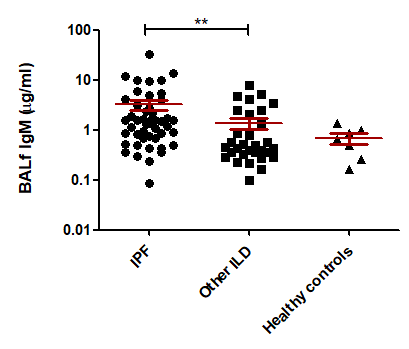


B

C

D


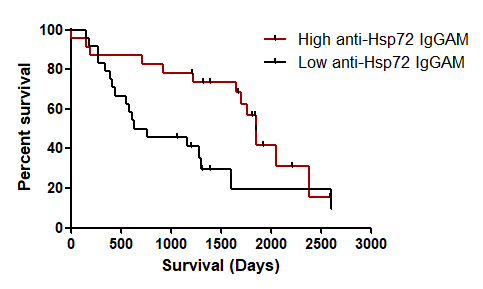

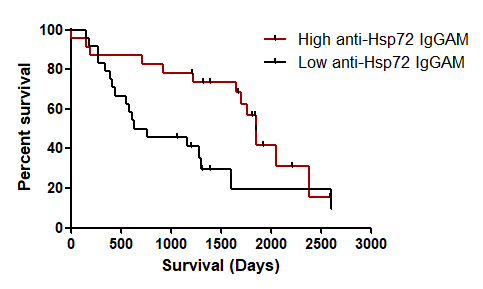


Supplementary figure 3: Serum IgM had no difference between all ILD patients and healthy controls (p=0.30). BALf IgM was significantly elevated in IPF patients compared to other ILD patients (p=0.003). Serum (B, HR=1.03, 95% CI 0.58-1.82) and BALf (D, HR 0.99, 95% CI 0.49-2.02) IgM levels were not associated with IPF patient outcomes or survival.
