# Supplementary material for: Intrapulmonary Autoantibodies to HSP72 Are Associated with Improved Outcomes in IPF
Source: J Immunol Res. 2019 Apr 11;2019:1845128. doi: 10.1155/2019/1845128 (PMC6487088; doi:10.1155/2019/1845128)
Supplement: Supplementary 5 — Supplementary Figure 4: Hsp72-IgG complexes induce CXCL8 secretion from in vitro MDM culture. MDM cell culture supernatant was analysed for CXCL8 (a, c) and CCL18 (b, d) by ELISA after treatment with 100 ng/ml Hsp72, 1 μg/ml anti-Hsp72 IgG, 1 μg/ml Hsp72-IgG complex, or 50 ng/ml LPS ((a, b) n = 7) for 24 hours. Elevated CXCL8 secretion was seen in response to 10 μg/ml mouse monomeric Hsp72 IgG and Hsp72-IgG complexes ((a) p = <0.05 and p = <0.001, respectively). No secretion of CCL18 was seen in response to mouse anti-Hps72 IgG ((b) p = 0.24, LPS excluded). (c) and (d) show the measured concentrations of CXCL8 and CCL18 after MDM culture with 1 μg/ml isolated BALf IgG with and without preculture with 100 ng/ml Hsp72. A significant difference was observed in CXCL8 ((c) p = 0.012) and CCL18 ((d) p = 0.032) secretion; however, poststats failed to identify the difference which is likely due to the experiment being underpowered. CXCL8 secretion was induced by BALf IgG preincubated with 100 ng/ml Hsp72 in a nonsignificant trend compared to BALf IgG alone ((c) p = 0.063). [file 1845128.f5.docx]

Mouse Anti-Hsp72 antibodies induce CXCL8 secretion from MDMs. Paired experiments using a commercial anti-Hsp72 antibody (mouse monoclonal IgG1) with LPS control. Macrophages in these experiments were cultured from isolated blood monocytes and stimulated with monomeric (anti-Hsp72) and complexed to Hsp72 (Hsp72-IgG) anti-Hsp72 antibodies. Experiments with the mouse antibodies showed that monomeric and complexed anti-Hsp72 antibodies induced CXCL8 secretion, but not CCL18 (no secretion of TGF-β or IL-10 was seen in these experiments either, data not shown). Further statistics have been done on the concentrations of isolated BALf IgG (C and D), it should be noted these experiments were set up as paired tests and are not sufficiently powered for meaningful interpretation.


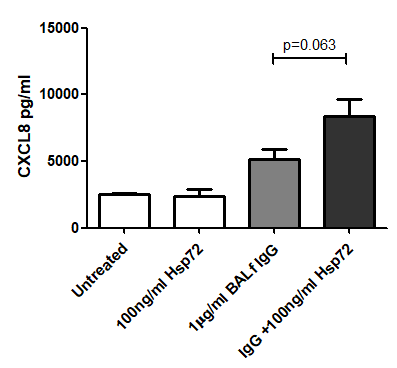

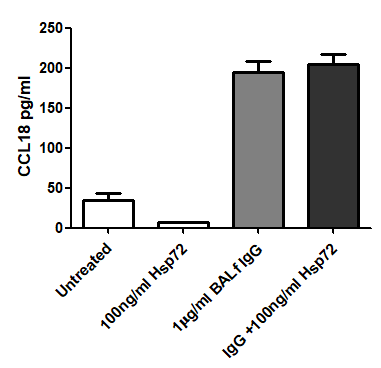

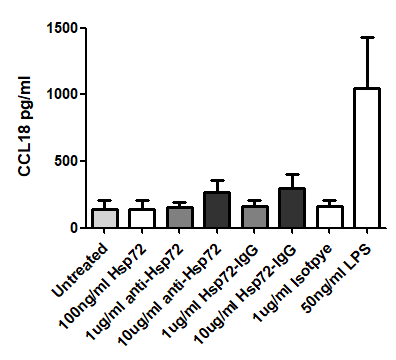

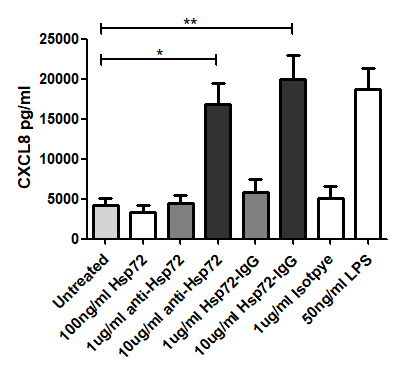


A

B

C

D

Supplementary figure 4. Hsp72-IgG complexes induce CXCL8 secretion from *in vitro* MDM culture. MDM cell culture supernatant was analysed for CXCL8 (A and C) and CCL18 (B and D) by ELISA after treatment with 100ng/ml Hsp72, 1μg/ml anti-Hsp72 IgG, 1μg/ml Hsp72-IgG complex or 50ng/ml LPS (A and B, n=7) for 24 hours. Elevated CXCL8 secretion was seen in response to 10ug/ml mouse monomeric Hsp72 IgG and Hsp72-IgG complexes (A, p=<0.05 and p=<0.001 respectively). No secretion of CCL18 was seen in response to mouse anti-Hps72 IgG (B, p=0.24, LPS excluded). Figures C and D show the concentrations measured of CXCL8 and CCL18 after MDM culture with 1µg/ml isolated BALf IgG with and without pre-culture with 100ng/ml Hsp72. A significant difference was observed in CXCL8 (C, p=0.012) and CCL18 secretion (D, p=0.032), however post-stats failed to identify the difference which is likely due to the experiment being underpowered. CXCL8 secretion was induced by BALf IgG pre-incubated with 100ng/ml Hsp72 in a non-significant trend compared to BALf IgG alone (C, p=0.063).
