# Supplementary material for: Intrapulmonary Autoantibodies to HSP72 Are Associated with Improved Outcomes in IPF
Source: J Immunol Res. 2019 Apr 11;2019:1845128. doi: 10.1155/2019/1845128 (PMC6487088; doi:10.1155/2019/1845128)
Supplement: Supplementary 6 — Supplementary Figure 5: the in-house optimised and commercial ELISA had a correlation in anti-Hsp72 antibody detection in the BALf. Serum concentrations of anti-Hsp72 antibodies did not correlate between the two ELISAs ((a) p = 0.24, Spearman r = 0.16). BALf concentrations were used normalised to total IgG as nonnormalised data for anti-Hsp72 IgGAM is unavailable. BALf concentrations of anti-Hsp72 antibodies correlated between ELISAs ((b) p = 0.0009, Spearman r = 0.48). Anti-Hsp72 IgG concentrations not normalised to total IgG had a near significant correlation with total IgG normalised anti-Hsp72 IgGAM concentrations (not shown, p = 0.051, Spearman r = 0.30). [file 1845128.f6.docx]

To compare the anti-Hsp72 antibody ELISAs used, results were compared in samples that were used in both ELISAs (using a Spearman Rank analysis). In the serum no correlation was observed between the ELISAs, but in the BALf a positive correlation was observed. It is unclear why this difference is observed but to speculate this may be due to the presence of anti-Hsp72 IgA or IgM in the serum. Alternatively, there is an elevated presence of Hsp72 in the serum but not the BALf, the impact this may have on the presence and retention or lack of with Hsp72 antibodies prior to clearance by immune cells when comparing the serum and BALf is unknown in this context.


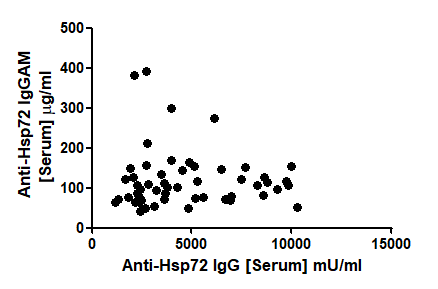

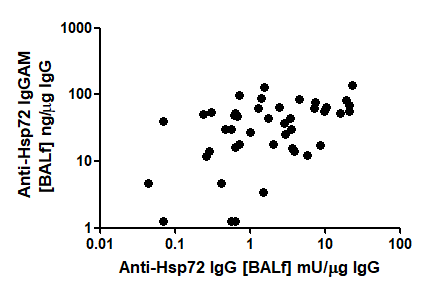


A

B

Supplementary figure 5. The in-house optimised and commercial ELISA had a correlation in anti-Hsp72 antibody detection in the BALf. Serum concentrations of anti-Hsp72 antibodies did not correlate between the two ELISAs (A, p=0.24, Spearman r=0.16). BALf concentrations were used normalised to total IgG as non-normalised data for anti-Hsp72 IgGAM is unavailable. BALf concentrations of anti-Hsp72 antibodies correlated between ELISAs (B, p0.009, Spearman r=0.48). Anti-Hsp72 IgG concentrations not normalised to total IgG had a near significant correlation with total IgG normalised anti-Hsp72 IgGAM concentrations (not shown, p=0.051, Spearman r=0.30).
